# New Advances in Titanium-Mediated Free Radical Reactions

**DOI:** 10.3390/molecules171214700

**Published:** 2012-12-11

**Authors:** Bianca Rossi, Simona Prosperini, Nadia Pastori, Angelo Clerici, Carlo Punta

**Affiliations:** 1 Department of Chemistry, Materials, and Chemical Engineering “Giulio Natta”, Politecnico di Milano, P.zza Leonardo da Vinci 32, 20133 Milano, Italy; E-Mails: brossi@chem.polimi.it (B.R.); simona.prosperini@chem.polimi.it (S.P.); nadia.pastori@polimi.it (N.P.); angelo.clerici@polimi.it (A.C.); 2 INSTM (National Consortium for Materials Science and Technology) Local Unit, Politecnico di Milano, 20133 Milan, Italy

**Keywords:** radical chemistry, titanium, titanocene, TiCl_3_, TiCl_4_, nucleophilic addition, pinacolization, cross-coupling, ring opening of epoxides, multicomponent reactions

## Abstract

Titanium complexes have been widely used as catalysts for C-C bond-forming processes via free-radical routes. Herein we provide an overview of some of the most significant contributions in the field, that covers the last decade, emphasizing the key role played by titanium salts in the promotion of selective reactions aimed at the synthesis of multifunctional organic compounds, including nucleophilic radical additions to imines, pinacol and coupling reactions, ring opening of epoxides and living polymerization.

## 1. Introduction

Since the pioneering work of Barton *et al.* [1] of 1960, the potential of radical reactions has been extensively explored, thanks also to the growth of advanced techniques for the detection of radicals and the definition of their structure and reactivity. This ongoing research has allowed the development of innovative selective free-radical reactions, occurring under mild conditions and tolerated by common functional groups, making such processes suitable for the synthesis and functionalization of organic compounds [2,3]. In the last decade, important improvements in this filed have been achieved by combining radical chemistry with transition-metal catalysis, due to the key role of transition metal complexes both in promoting and highly controlling sophisticated free-radical synthetic routes [4,5,6,7]. This approach has been adopted for different applications, ranging from oxidative [8,9,10] and reductive [11] radical processes to the selective C-C and C-N bond formation [12,13,14,15,16,17,18,19], passing through living polymerization [20,21,22].

Among the transition metals widely employed for these purposes, in the present review we focus on titanium and address the recent advances in selective free-radical processes mediated by Ti(III) and Ti(IV) complexes. Titanium, the seventh most abundant metal on Earth, is one of the cheapest transition metals and a lot of titanium compounds are nontoxic and environmentally friendly. The high added value of using titanium salts to promote radical synthetic routes not only relies on their selective intervention in the initiation and the termination steps of the radical chain, but also on their remarkable coordinating role shown, due to their Lewis acid character.

Limiting our overview to the results reported in the literature in the past twelve years, Section 2 describes the potential of Ti(III)/PhN_2_^+^ and Ti(III)/hydroperoxide systems in promoting one-pot, multicomponent, nucleophilic radical additions to aldimines and ketimines generated *in situ*. Section 3 deals with the most significant examples achieved in pinacol and coupling reactions while, in Section 4, ample discussion is devoted to the ring-opening of epoxides mediated and catalyzed by titanocenes. Finally, in Section 5, we briefly disclose the recent results reported in the field of living polymerization.

It is noteworthy that titanium dioxide is also able to promote free-radical processes by photoactivation at suitable wavelengths, but this chemistry is not the object of the present review. The combined action of light and TiO_2_, under oxidative conditions, finds interesting applications in the field of pollutant photodegradation, selective organic photosynthesis and antibacterial activity. For a comprehensive overview on the TiO_2_ photocatalytic activity, we refer the reader to some very recent reviews [23,24,25,26].

## 2. Nucleophilic Radical Addition to Imines

Nucleophilic radical addition to imines, iminium salts or hydrazones, mediated by transition metal salts such as manganese [27], copper [28,29,30], and zirconium [31,32,33], represents a valuable alternative to the classical ionic routes for the selective C-C bond formation and the synthesis of high added value polifunctional molecules [12,13,14,15,16,17]. This approach becomes even more intriguing if the transition metal is able to promote, in one-pot, the *in situ* formation of the imine followed by nucleophilic addition, in accordance with the multicomponent reaction (MCR) strategy. In the last decade TiCl_3_, because of this particular specificity, has attracted the increasing interest of our research group, enabling us to develop quite a number of free-radical MCRs.

### 2.1. TiCl_3_/ArN_2_^+^ System

**Scheme 1:**
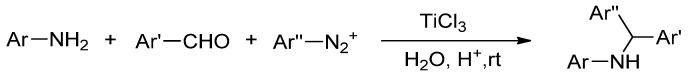
Arylative amination of aldehydes promoted by the TiCl_3_/ArN_2_^+^ system.

The selective arylative amination of aromatic aldehydes using the TiCl_3_/ArN_2_^+^ system (Scheme 1) was first reported by Clerici and Porta in 1990 [34]. According to this procedure, Ti(III) promotes the aryldiazonium salt decomposition to an aryl radical. Simultaneously, the Ti(IV) formed, due to its oxophilicity, favors the *in situ* formation of aldimines from anilines and aldehydes. Furthermore, as a strong Lewis acid, Ti(IV) enhances, via N-complexation, the electrophilicity of the C=N bond carbon atom towards nucleophilic attack by the aryl radical.

**Scheme 2:**
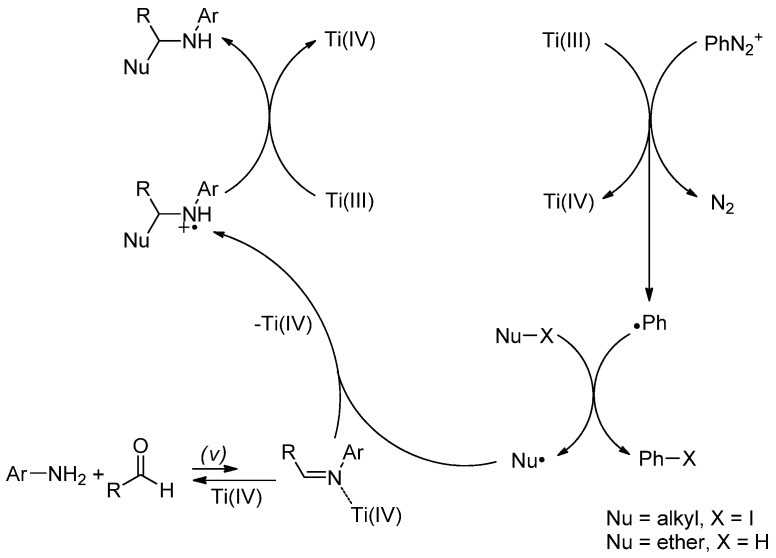
Nucleophilic radical addition to aldimines promoted by the TiCl_3_/PhN_2_^+^ system.

This reaction occurs in an aqueous environment, where it is well known that imines, easily hydrolysable, are quite unstable and less prone to a nucleophilic attack. Later, in 2005, this approach was successfully extended to a wider range of nucleophilic radical sources by introducing a fourth component in the cascade reaction. In fact, in the presence of alkyl iodides [35], the phenyl radical, deriving from the corresponding diazonium salt decomposition, promotes the fast iodide-atom abstraction, generating an alkyl radical which, in turn, undergoes a nucleophilic attack onto aldimines generated *in situ* (Scheme 2).

According to a similar procedure, the nucleophilic addition of ethers onto aldimines was performed via *α*-H atom abstraction via the same phenyl radical source (Scheme 2) [36], providing the first radical version of the Mannich reaction. The resulting iminium radical is then reduced by a second equivalent of TiCl_3_, rendering the overall mechanism irreversible towards the formation of the desired product.

### 2.2. TiCl_3_/ROOH and TiCl_4_-Zn/ROOH Systems

**Scheme 3:**
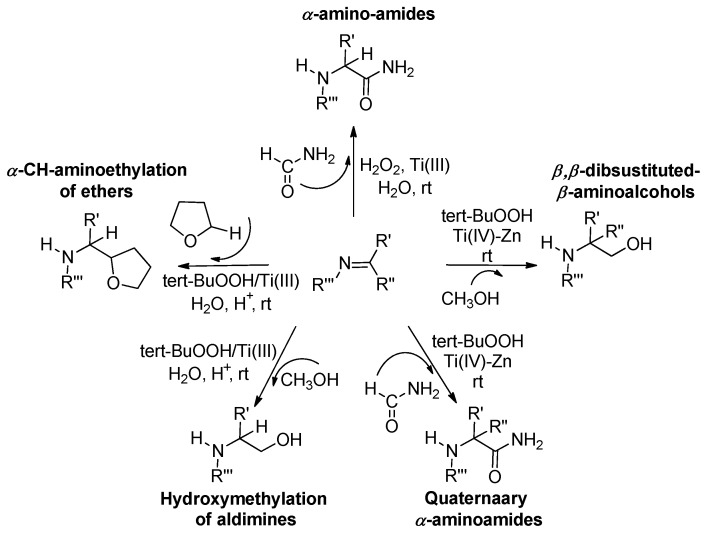
Radical addition to imines triggered by TiCl_3_ or TiCl_4_-Zn/ROOH systems.

In 2006, we reported a series of new one-pot multicomponent reactions mediated by TiCl_3_ or TiCl_4_-Zn hydroperoxide systems, which overcome some limits of the previously disclosed approach. The choice of the right hydroperoxide allows the formation of more practical, efficient and selective precursors of the radical cascade. As a consequence, the new procedure can be extended to a wider range of nucleophilic sources and can be applied even onto ketimines generated *in situ*, allowing us to create different building blocks for bioactive molecules (Scheme 3). Moreover, this reaction occurs under mild conditions in 30–60 min. Finally, the proposed approach requires neither the pre-formation of the imine nor the protection of the amino group and may easily be conducted under aqueous conditions.

#### 2.2.1. TiCl_3_/ROOH

**Scheme 4:**
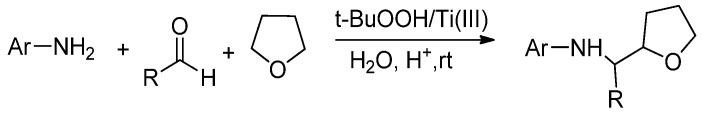
Aminoalkylation of ethers promoted by the TiCl_3_/t-BuOOH system.

An alternative route to the radical aminoalkylation of ethers was proposed by Porta and co-workers in 2006 [37]. The TiCl_3_-*tert*-butylhydroperoxide (*t*-BuOOH) system was revealed to be a more practical, efficient and selective precursor of *α*-alkoxyalkyl radicals from ethers. Accordingly, TiCl_3_/*t*-BuOOH readily assemble an aniline, an aldehyde, and an ether, leading to 1,2-aminoethers (Scheme 4).

**Scheme 5:**
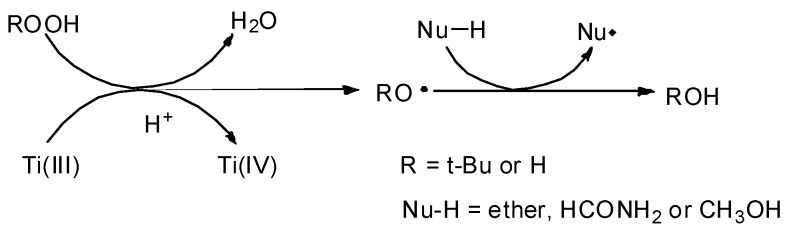
Nucleophilic radical generation promoted by the TiCl_3_/t-BuOOH system.

In agreement with the general proposed mechanism (Scheme 5), the dropwise addition of TiCl_3_ into the reaction medium promotes the decomposition of the hydroperoxide, leading to the formation of *tert*-butoxyl radical (*t*-BuO·). The latter undergoes an *α*-H abstraction from the ether, with higher selectivity with respect to the phenyl radical generated by the TiCl_3_/PhN_2_^+^ system (no *β*-hydrogen abstraction was observed). Once formed, the nucleophilic ketyl radical adds to the aldimine generated *in situ*, in analogy with the mechanism indicated in Scheme 2.

**Scheme 6:**
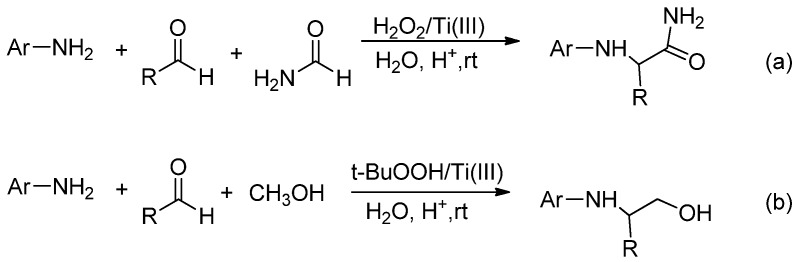
(**a**) Carbamoylation of aldimines. (**b**) Hydroxymethylation of aldimines.

The reaction goes like a titration and can be easily monitored by considering the different color of the species involved: Ti(III) (violet) and Ti(IV) (orange). Thus, the addition of the TiCl_3_ solution stops when a pale blue color is barely maintained to ensure the complete decomposition of the peroxide. This methodology was then extended to the selective carbamoylation of aldimines in formamide [38], providing a radical version of the Strecker synthesis of *α*-amino amides (Scheme 6a), and to the aminoalkylation of methanol [39], leading to the formation of *α*,*β*-amino alcohols (Scheme 6b).

**Scheme 7:**
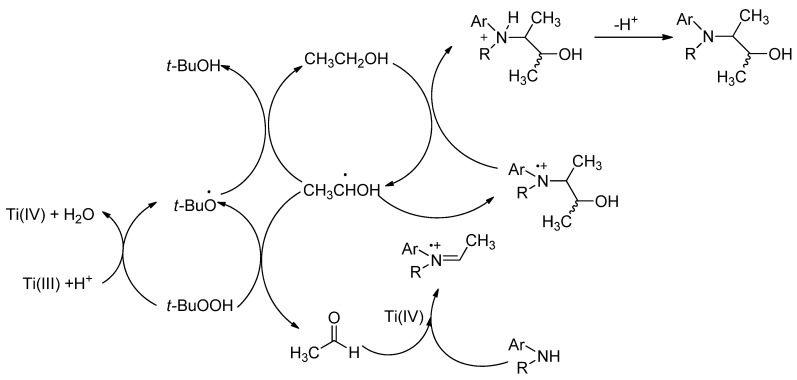
Domino reaction in alcoholic solvent promoted by the TiCl_3_/t-BuOOH system.

When the same protocol is used in the presence of an alcoholic solvent different from methanol, a domino reaction competes with the one-pot process (Scheme 7) [40].

In fact, in this case the ketyl radical formed behaves as a strong reducing agent [41] and competes with Ti(III) in the reduction of *t*-BuOOH, leading to the formation of the corresponding aldehyde. The latter, when in the presence of an aniline, promotes the formation of an aldimine, which is prone to nucleophilic attack by a second ketyl radical. As a consequence, when the reaction is carried out in the absence of aldehydes, it proceeds to the formation of β-amino alcohols by *in situ* generation of an aldehyde.

#### 2.2.2. TiCl_4_-Zn/ROOH

**Scheme 8:**
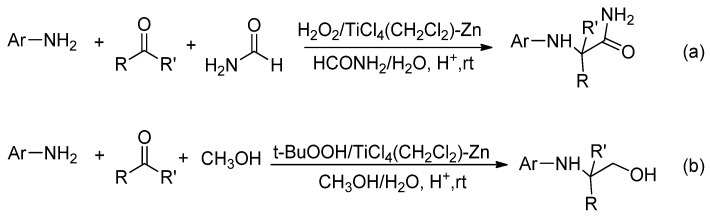
(**a**) Carbamoylation of ketimines. (**b**) Hydroxymethylation of ketimines.

Since ketimines are less stable in aqueous medium and less reactive than aldimines, studies involving the reductive radical addition to ketimines are scant in comparison with those conducted on their aldimine counterparts. Moreover, in almost all cases the preformation of ketimines is required. It is because of this low stability under non-anhydrous conditions that the TiCl_3_/ROOH system resulted ineffective when an aniline, a ketone and formamide were assembled in one-pot with the intention of promoting the formation of quaternary *α*-amino amides. Nevertheless, we recently reported an optimization of the previously discussed protocol, consisting in the replacement of the aqueous TiCl_3_ with an anhydrous solution of TiCl_4_ in CH_2_Cl_2_ in combination with Zn powder (Scheme 8a) [42]. The same protocol was successfully applied in methanol solvent for the selective hydroxymetylation of ketimines, affording the corresponding *β*,*β*-disubstituted-*β*-amino alcohols (Scheme 8b) [43].

**Scheme 9:**
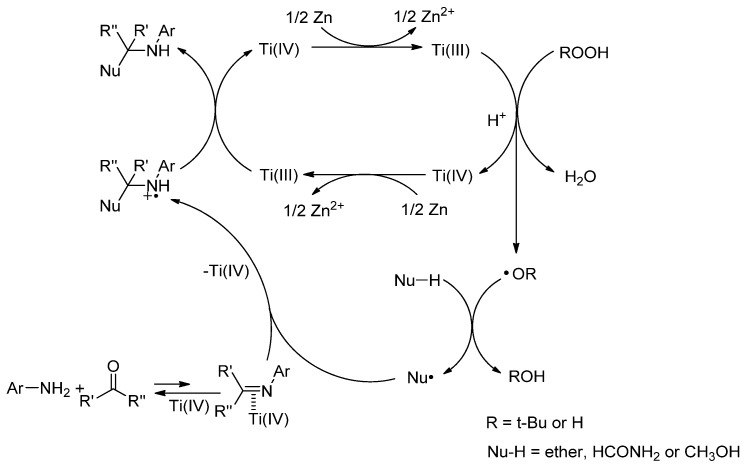
Nucleophilic addition to ketimines promoted by the TiCl_4_-Zn/ROOH system.

The mechanism is analogous to that previously discussed and Zn metal has the unique role of sacrificial reductant for the in situ generation of Ti(III) from Ti(IV) (Scheme 9).

**Scheme 10:**
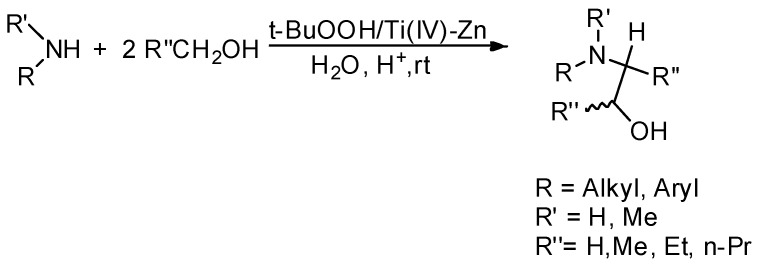
Domino reaction in alcohols promoted by the TiCl_4_-Zn/ROOH system.

Surprisingly, the TiCl_4_-Zn/*t*-BuOOH combination was also revealed to be even more effective and selective with respect to the TiCl_3_/*t*-BuOOH approach in promoting domino reactions. 1,2-Amino alcohols were obtained in high yields by simply combining two molecules of ethanol or propanol in the presence of aromatic and aliphatic amines, without requiring the addition of aldehydes or ketones (Scheme 10) [44]. Even more interesting is that an analogous reaction occurred by operating in cyclic ether solvents [44].

**Scheme 11:**
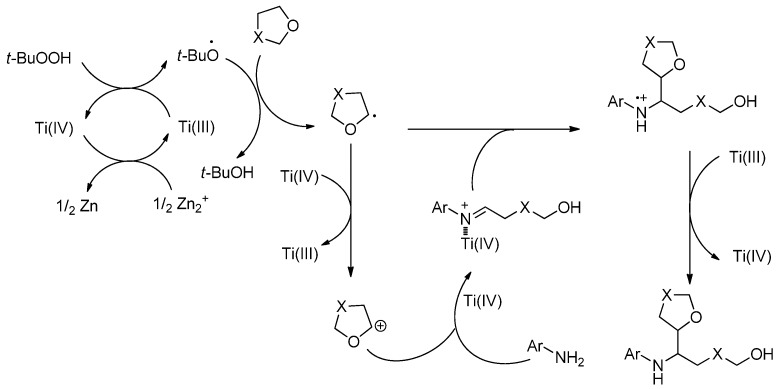
Domino reaction in cyclic ethers promoted by the TiCl_4_-Zn/ROOH system.

According to the proposed mechanism (Scheme 11), it was possible to synthetize amino alcohols by electrophilic amination of ketyl radicals, promoting the formation of the imine via ring opening of the ether cyclic and the subsequent nucleophilic radical addition by a second ketyl radical.

### 2.3. Cp_2_TiCl_2_/Zn Systems

**Scheme 12:**
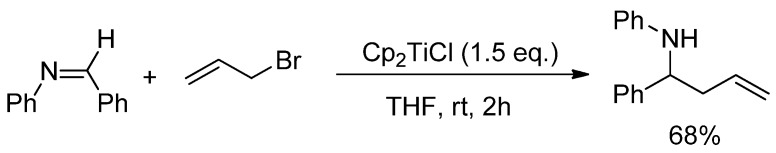
Cp_2_TiCl-mediated allylation of aromatic aldimines.

In 2011, Saha and Roy reported a selective radical-induced allylation of pre-formed aldimines for the synthesis of homoallyl amines [45]. The combination of titanocene(IV) dichloride and Zn dust in THF under argon leads to the formation of Cp_2_TiCl in situ. The latter acts as radical initiator, promoting the formation of the allyl radical from the corresponding allyl bromide (Scheme 12). This approach was successfully applied to the synthesis of deoxy aza-disaccharides and for the preparation of the bicyclic skeleton of alkaloids.

## 3. Coupling Reactions

Radical coupling mediated by transition metal salts represents one of the pillars of free-radical synthesis. Once again, simple titanium salts and titanium complexes bearing specific organic ligands result particularly effective in promoting intramolecular as well as intermolecular coupling with high stereo-, diastero- and even enantioselectivity [46,47]. Among the several protocols developed, pinacolization, allylation and cross-coupling reactions have attracted particular attention in the last decade.

### 3.1. Pinacol Reactions

The pinacolization of aldehydes is one of the most studied reactions in the area of radical coupling for C-C bond formation. This interest arises from the usefulness of hydrobenzoins as chiral auxiliaries and ligands for stereoselective organic synthesis. Moreover, the pinacol reaction represents an ideal tool for studying the enatioselective catalyzed formation of radicals [48].

**Scheme 13:**
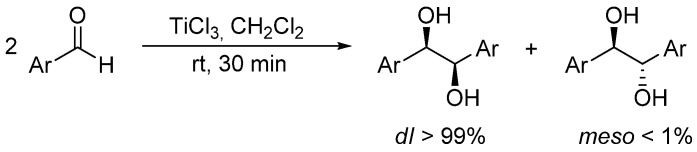
*dl*-Stereodirecting pinacolization of aromatic aldehydes promoted by TiCl_3_.

This reaction can be accomplished with a variety of one- or two-electron metal reducing agents. Our group has widely investigated the role that TiCl_3_ and TiCl_4_ play in promoting pinacolization of aromatic aldehydes. In particular, a *dl*-stereodirecting pinacolization was accomplished by using TiCl_3_ in a strictly anhydrous non-coordinating solvent (CH_2_Cl_2_), favored by Ti(III)-complexation of the oxygen atom of the carbonyl group, which lowers the reduction potential of the aromatic aldehyde (Scheme 13) [49].

Later on, in the same CH_2_Cl_2_ solvent, an analogous reactivity was observed when TiCl_4_ is coupled with diisopropylethylamine (DIPEA). Porta and co-workers [50] revealed how, in this case, the TiCl_4_/DIPEA complexation is the driving force of the overall process, causing the instantaneous formation of Ti(III) by an inner-sphere electron transfer from Ti(IV) to DIPEA. Once generated, Ti(III) coordinates the carbonyl oxygen of an added aromatic aldehyde, promoting its reductive dimerization.

More recently we have unexpectedly found that, despite the non-anhydrous conditions, the aqueous TiCl_3_/*t*-BuOOH redox system promotes the hydrodimerization of aromatic aldehydes, when used in combination with an alcoholic co-solvent like ethanol [41].

**Scheme 14:**
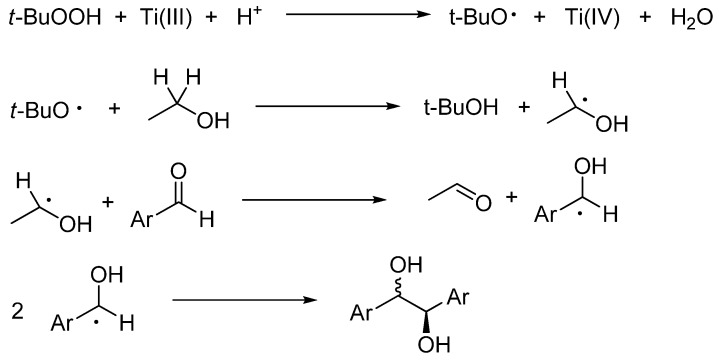
Proposed mechanism for the TiCl_3_/*t*-BuOOH mediated pinacolization.

In accordance with the proposed mechanism (Scheme 14), the explanation of the paradox relies in the reducing power of the *α*-hydroxyalkyl radicals generated *in situ* via H-atom abstraction from the alcoholic solvent by *tert*-butoxy radical. These ketyl radicals, instead of Ti(III), are the actual reductants of the aromatic aldehydes.

Pinacolization of aromatic ketones like acetophenone can be performed in good yields and high stereoselectivity (*dl*/*meso* ratio = 9/1) in the presence of Cp_2_TiCl, generated *in situ* by combining Cp_2_TiCl_2_ with an excess of Mn dust [51].

**Figure 1:**
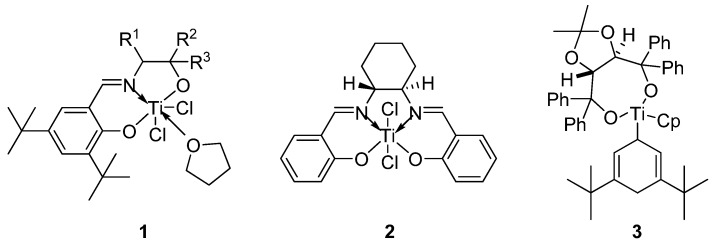
Chiral Ti(IV) complexes.

Moreover, new families of Ti(IV) complexes bearing chiral ligands (**1**, **2** and **3**, Figure 1) have been developed in the last decade. These derivatives, when employed in combination with co-reducing agents, revealed to be ideal precursors of chiral Ti(III) catalysts for the enantioselective pinacol coupling of aldehydes.

**Scheme 15:**
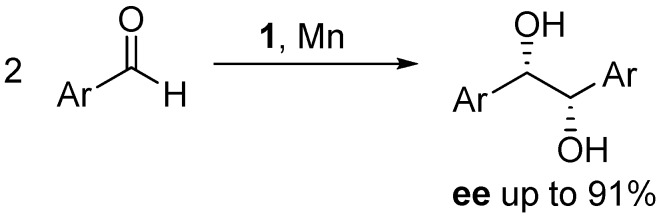
Riant’s pinacolization mediated by stoichiometric amounts of complex **1**.

In 2001 Riant and co-workers [52] reported the combined use of stoichiometric amounts of the titanium-Schiff base complex **1** and Mn to promote the asymmetric *dl*-diastereoselective pinacolization with high yields and enantioselectivity up to 91% (Scheme 15).

**Scheme 16:**
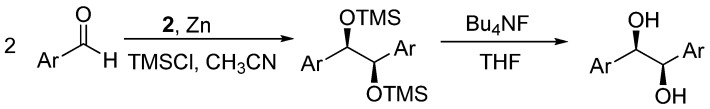
Pinacolization mediated by catalytic amounts of complex **2**.

An analogous result was achieved by Joshi *et al.*, who succeeded in performing a catalytic cycle in CH_3_CN with 10 mol % concentration of complex **2** and stoichiometric amounts of Me_3_SiCl (TMSCl), followed by the addition of tetrabutylammonium fluoride in THF (Scheme 16) [53].

**Scheme 17:**
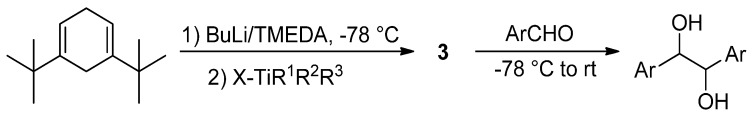
Pinacolization of aromatic aldehydes promoted by **3**.

In 2005, Knoop and Studer reported a new method for the *in situ* generation of Ti(III) complexes without requiring a co-reducing reagent [54]. Cyclohexadienyl-Ti(IV) compounds, prepared from the corresponding lithiated cyclohexadienes, led to the corresponding Ti(III) complexes upon thermal C-Ti bond homolysis (Scheme 17). Following this procedure, complex **3** was readily prepared and employed to perform pinacol coupling of aromatic aldehydes with diastereomeric and enantiomeric excess.

### 3.2. Allylation Reactions

**Scheme 18:**
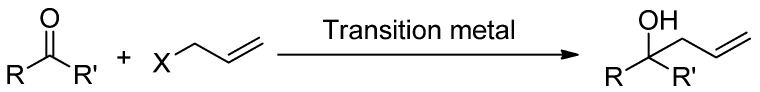
Barbier-type allylation of carbonyl derivatives.

Among radical coupling reactions, the Barbier-type allylation of carbonyl derivatives, mediated by different transition metals, has attracted particular attention due to its considerable synthetic relevance (Scheme 18). The generic strategy for this process consists in the transformation of allyl halides into the corresponding allyl radicals which, in turn, would add onto the carbonyl compounds present in the reaction medium.

Very recently, Justicia *et al.* have shown that an excess of titanocene(III) chloride, generated *in situ* by the Cp_2_TiCl_2_/Mn-dust system, allows to extend this protocol to the crotylation of carbonyl compounds [55].

**Scheme 19:**
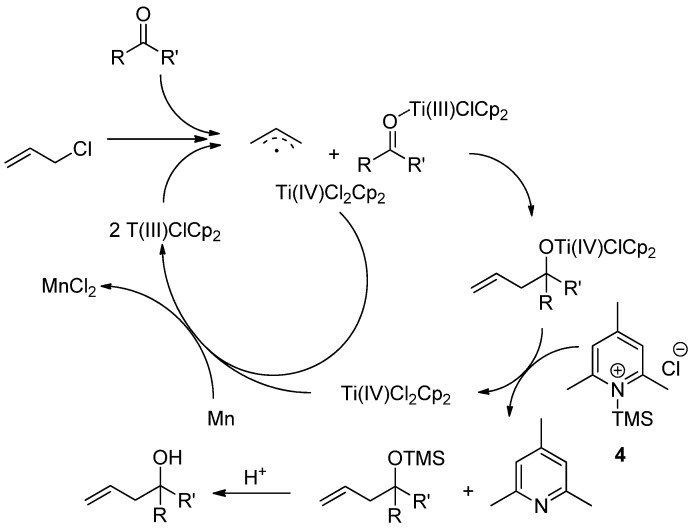
Barbier-type allylation mechanism promoted by catalytic titanocene.

However, most of the reported protocols usually require stoichiometric amounts of these metals, which is detrimental for the sustainability of the process. Stoichiometric quantities are generally necessary even when operating in the presence of Ti complexes, limiting the applicability of this approach to enatioselective additions [46]. In this context, in 2009 a comprehensive contribution by Oltra and co-workers has shown how catalytic proportions of titanocene(III) complexes, when combined with the regenerating agent **4** (obtained by mixing TMSCl and 2,4,6-collidine), are able to promote the Barbier-type allylation, in accordance with the mechanism reported in (Scheme 19) [56].

**Scheme 20:**
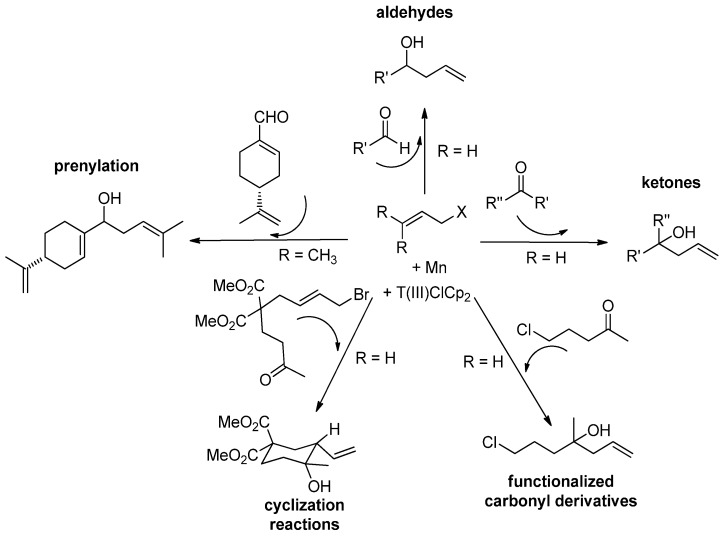
Examples of Barbier-type allylations promoted by catalytic titanocene.

This approach has been successfully applied for the allyl radical addition onto aldehydes and ketones (including functionalized carbonyl substrates), for cyclization reactions and for prenylation of aldehdyes (Scheme 20).

The titanocene(III)-mediated Barbier-type prenylation to α,β-unsaturated aldehydes, which allows the introduction of hydroxyl groups at desiderable positions, and the subsequent radical cyclization promoted by the same titanium complex, open the straightforward access to polyhydroxylated terpenoids [57].

**Scheme 21:**
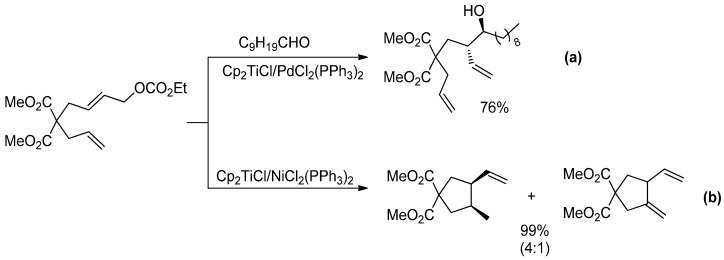
Divergent Ti-mediated allylations with modulation by Palladium or Nickel.

The main limitation of the Barbier-type approach is that it requires the use of allylic halides as reactive substrates, while allylic carbonates and carboxylates, which are easily prepared and handled, are inert against titanocene(III) complexes. To overcome this limit, Oltra and co-workers proposed the combination of Cp_2_TiCl with palladium and nickel complexes [58]. Indeed, it is well known that these metal derivatives are able to activate allylic carbonates and carboxylates by forming η^3^-allylmetal complexes. The authors have demonstrated how the choice of the metal is crucial in modulating the Cp_2_TiCl activity in the allylation reaction. In fact, while palladium salts promote the intermolecular coupling with electrophilic reagents (Scheme 21a), the intramolecular allylation of alkenes with allylic carbonates occurs in the presence of nichel complexes, leading to carbocylic derivatives (Scheme 21b).

**Scheme 22:**
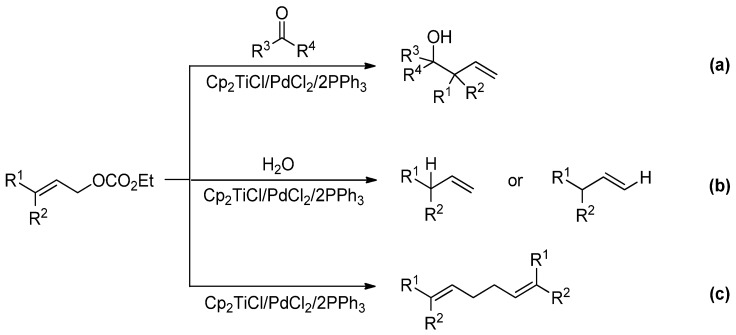
Titanium/Palladium mediated reactions.

**Scheme 23:**
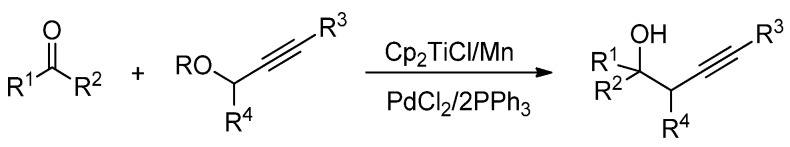
Titanium/palladium-mediated propargylation of ketones.

**Scheme 24:**
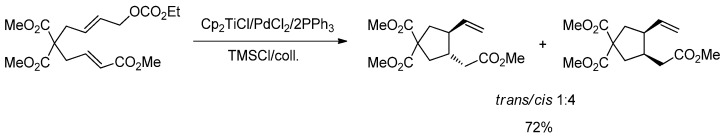
Titanium/palladium-mediated intramolecular Michael-type addition.

The titanium/palladium-mediated system has been successfully applied by the same research group for the selective allylation, crotylation and prenylation of aldehydes and ketones [59] (Scheme 22), for the synthesis of homopropargyl alcohols using propargyl carbonates as pronucleophiles [60] (Scheme 23), and to promote the intramolecular Michael-type addition of allylic carboxylates to activated alkenes [61] (Scheme 24). More recently, the efficient allylation of carbonyl compounds starting with allyl carbonates has been performed by replacing Pd with Ni [62].

### 3.3. Other Coupling Reactions

Several other coupling reactions promoted by titanium complexes have been recently reported. Roy and co-workers have proposed the use of the Cp_2_TiCl_2_/Zn-dust system to promote the synthesis of furan derivatives such as (±)-evodone from *α*-bromo-*β*-keto enolethers [63]. The same approach has been successfully applied to the asymmetric synthesis of *α*-methylene bis-*γ*-butyrolactone [64].

**Scheme 25:**
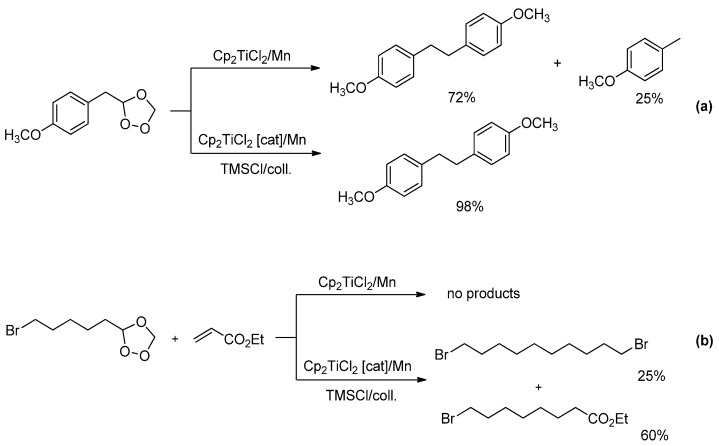
Homocoupling versus cross-coupling in the Ti-catalyzed opening of ozonides.

The Cp_2_TiCl-catalyzed homolytic opening of ozonides, reported by Rosales *et al.* [65], provides carbon centered radicals suitable to form C-C bonds via both homocoupling and cross-coupling processes (Scheme 25).

**Scheme 26:**
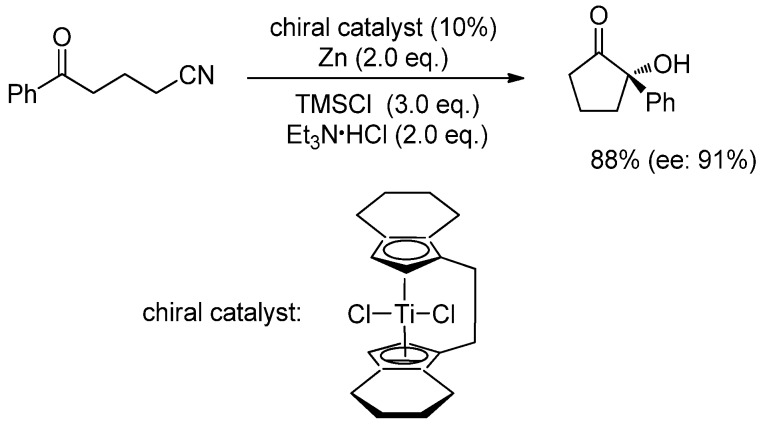
Reductive cyclization of ketonitriles.

Very recently, Streuff and co-workers have reported an efficient enatioselective reductive cyclization of ketonitriles promoted and catalyzed by chiral Ti(III) catalysts (Scheme 26) [66].

An example of the potential of radical chemistry is the use of the Ingold-Fisher “Persistent Radical Effect” (PRE) which has found ample employment in cross-coupling reactions for the selective synthesis of organic compounds. According to PRE, the cross-coupling between two radicals occurs when the two species are generated at similar rates and one is more persistent than the other, that is with a lifetime significantly greater than that for a methyl radical under the same conditions [67].

**Scheme 27:**
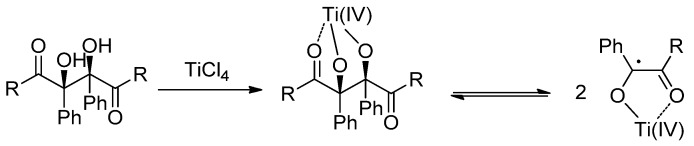
C-C bond cleavage of *α*,*β*-dihydroxy ketones promoted by TiCl_4_.

In 2008 we reported how *α*,*β*-dihydroxy ketones can be used as a source of stabilized radicals via selective C-C bond cleavage promoted by TiCl_4_ [68] the coordinating effect of the metal ion induces an increased stabilization onto the new radicals, driven by the captodative effect (Scheme 27).

**Scheme 28:**
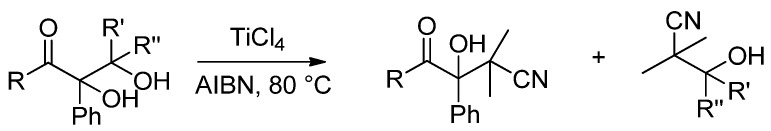
Synthesis of *β*-hydroxynitriles from *α*,*β*-dihydroxy ketones and AIBN.

This observation prompted us to develop a new one-pot multicomponent route leading to the synthesis of polyfunctional derivatives of high added value. The generation, in the same reaction medium, of transient radicals via thermal decomposition of 2,2′-azo-bis-isobutyronitrile (AIBN) yielded an innovative methodology for the synthesis of *β*-hydroxynitriles by cross-combination between Ti(IV)-stabilized radicals and the *α*-cyanoisopropyl radical (Scheme 28).

## 4. Epoxide Reactions Mediated by Titanocene

**Scheme 29:**
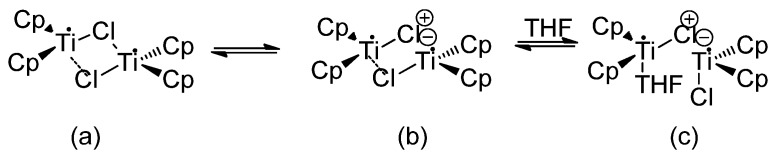
Structures of Cp_2_TiCl in solution: dimer (**a**), half-open dimer (**b**), reactive species binding the solvent (**c**).

Bis (cyclopentadienyl) titanocene III chloride (Cp_2_TiCl) and substituted titanocenes are well known titanium complexes able to generate regioselective radical epoxide opening through single electron transfer (ET), both in stoichiometric and catalytic amounts [69,70]. The method of radical generation combines both the Lewis acid catalysis and the radical chemistry with interesting advantages: the initial ET by the metal, tuning the acidity of the metal center, avoids SN-type reactions, while the ensuing radical reaction can be controlled by the metal complex and his ligand. Cyclic voltammetry and kinetic measurements have disclosed the structure and mechanism of epoxide opening by Cp_2_TiCl reagents in solution: the activation of Cp_2_TiCl_2_ species by Mn or Zn reducing metals in THF solution leads to a mixture composed by the monomer Cp_2_TiCl and the chlorine bridged dimer (Cp_2_TiCl)_2_, but the more reactive species is the half-open dimer [71,72] (Scheme 29).

Typical radical reactions mediated by titanocene reagents regard selective reduction and deoxygenation of epoxides, C-C bond formation processes such as cyclizations, and intermolecular additions to activated olefins [73,74].

It is worth noting that, as the reaction mechanism generally proceeds via the most substituted (*i.e.*, energetically most stable) radical, the regiochemistry is the opposite (and complementary) to that normally expected for conventional SN_2_-type epoxide openings with carbon nucleophiles or hydride reagents [73,74].

### 4.1. Epoxide Deoxygenation

Though epoxides are usually prepared from olefins, their deoxygenation for the generation of C=C double bonds finds ample application in highly oxidized substrates like carbohydrates and other natural derivatives [75,76]. The process proceeds in good to excellent yields, with exceptional chemo-selectivity.

It has been shown that the deoxygenation occurs when operating in the absence of radical acceptors and that it depends on the substitution pattern [77]. On the basis of these observations, Fernández-Mateos and co-workers have recently reported the selective synthesis of terpenoids starting from pinene-oxide derivatives [78].

**Scheme 30:**
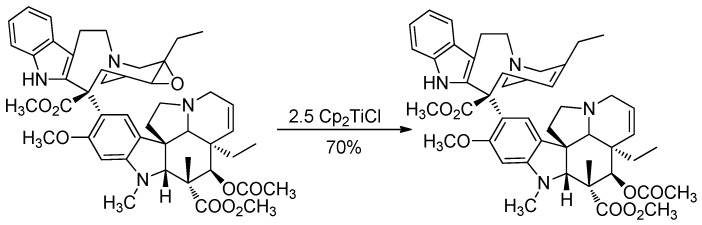
Deoxygenation of leurosine.

Doris and co-workers reported in 2002 the Cp_2_TiCl-mediated deoxigenation of the alkaloid leurosine (from *Catharantus roseus*), leading to anhydrovinblastine (Scheme 30), the key intermediate in the symthesis of the anticancer drug navelbine [79].

**Scheme 31:**
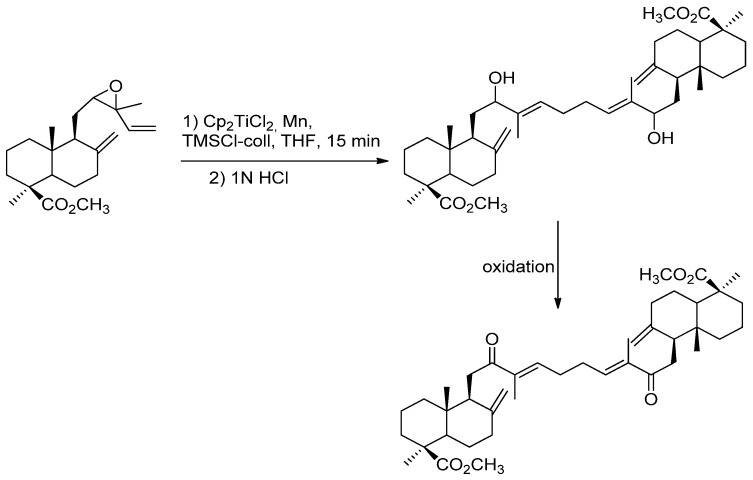
Dimerization of vinylepoxides for the preparation of homo-onecerans.

Furthermore, short and straightforward access to analogues of the natural onoceranes mediated by Cp_2_TiCl_2_ has been proposed by Barrero and his group [80]. The dimerization of vinyl-epoxides to 1,5-dienes occurs via generation of very stable *β*-titanoxy allylradicals. The latter undergo dimerization to homo-onoceranes, followed by oxidation (Scheme 31).

In 2009, Aldegunge *et al.* proposed the synthesis of alk-2-ene-1,4-diols by a new cascade-opening of 1,3-diepoxides, suggesting an alternative route to dihydroxytaxoids [81].

**Scheme 32:**
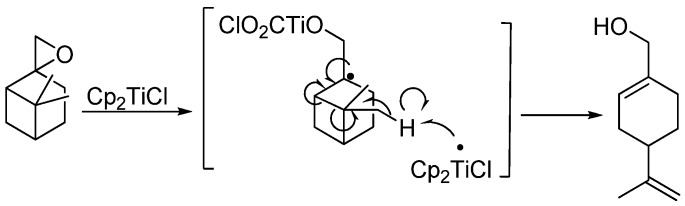
Disproportionation of epoxide through hydrogen atom transfer.

In the same year, Justicia et coworkers [82] showed that *β*-titanoxy radicals may rearrange to new trisubstituted radicals which undergo a mixed disproportionation process (via hydrogen atom transfer, Scheme 32) leading to allylic alcohols.

### 4.2. Reductive Epoxide Opening

The catalytic reductive epoxide opening via electron transfer from titanocenes has emerged as an attractive alternative to the classical nucleophilic substitution strategies. The titanium-mediated mechanism combines the well-established advantages of radical chemistry with a regioselectivity of ring opening opposite to that of nucleophilic substitutions, leading to a number of interesting and unusual applications in the synthesis of complex molecules [83,84,85,86,87].

#### 4.2.1. Synthesis of Alcohols

**Scheme 33:**
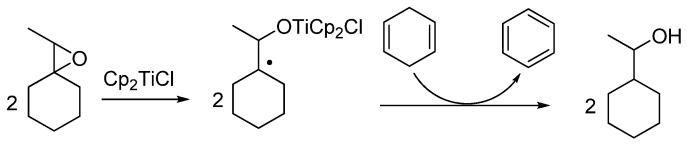
Titanocene-mediated reductive epoxide opening by 1,4-cyclohexadiene.

According to mechanistic studies, the depicted *β*-tytanoxyl radical epoxide complexes constitute the first intermediate of ring opening. These radicals are then reduced by hydrogen atom donor species, like for example 1,4-cyclohexadiene (Scheme 33).

If the use of catalytic amounts of titanocenes is attractive for reagent controlled radical reactions [88], on the other hand this process presents the disadvantage deriving from the generation of stoichiometric amounts of highly toxic reducing waste, such as benzene (Scheme 26). However, it has been demonstrated that titanocene reagents are able to activate water and methanol toward hydrogen atom transfer, by substantially lowering the bond dissociation energy (BDE) of the OH bond [89,90,91]. This combination is chemically more efficient and environmentally benign. Cp_2_TiCl-mediated reductive epoxide ring opening using water as a hydrogen source, allows access to alcohols with anti-Markovnikov regiochemistry from different epoxides. The use of D_2_O as a deuterium source, leads to an efficient synthesis of *β*-deuterated alcohols, which find successful application as internal standards in food analysis [92]. Another suitable hydrogen donor is H_2_ [93,94].

**Figure 2:**
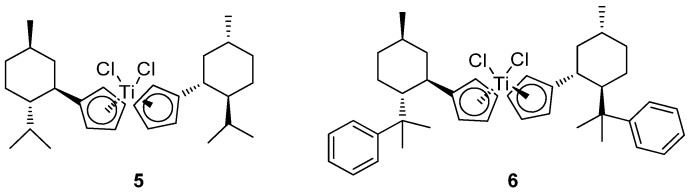
Different substitution patterns at the cyclopentadienyl ligand (Cp).

**Scheme 34:**
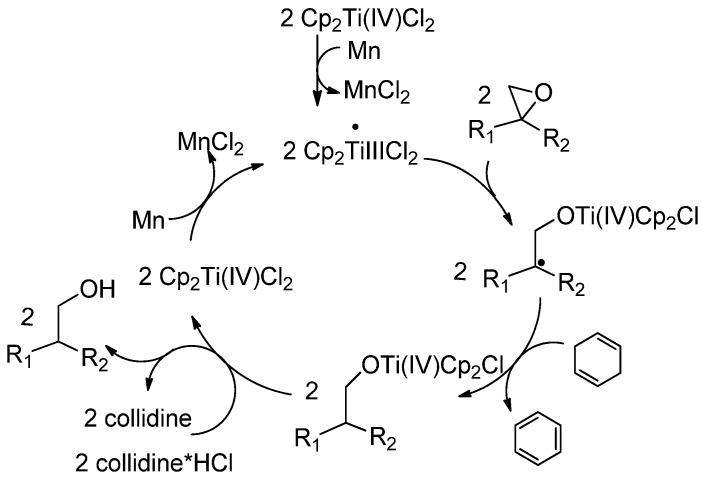
Mechanism of the titanocene-catalyzed reductive epoxide opening.

Titanocene-catalyzed procedure has been immediately extended by Gansäuer *et al.* to the enantioselective opening of *meso*-epoxides. Titanocene complexes with chiral ligands, such as those reported in Figure 2, were employed in combination with manganese dust and collidine hydrochloride, the former as a stoichiometric reductant and the latter having the role of regenerating the alcohol and titanocenes [95,96,97,98,99,100,101,102] (Scheme 34).

**Scheme 35:**
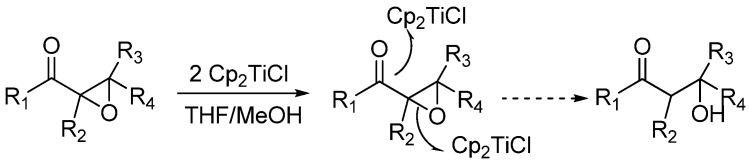
Example of elective reduction of *α*,*β*-epoxyketones.

By operating in the presence of collidinium hydrochloride, the catalytic reaction could be also performed in water. This approach was exploited for the selective reduction of aromatic ketones [103,104] and the selective synthesis of 2-methyl-1,3-diol frameworks, using THF as hydrogen donor [105]. Also *α*,*β*-epoxy ketones could be opened with Cp_2_TiCl, affording *β*-hydroxy ketones in a better fashion than with SmI_2_ (Scheme 35) [106].

The ring-opening reaction of tri-substituted epoxides promoted by Cp_2_TiCl on carvone derivatives has led to *exo*-methylene allylic alcohols as major compounds [107]. The generation of exocyclic olefins has been reported in the course of radical cyclizations of epoxy alkenes leading to monocyclic terpens as achilleol A [108], oxobicylic drimanes [109], eudesmanolides [110], and different ring synthons of paclitaxel [111]. In these cases, termination of the radical cycle is not reductive, but rather it involves *β*-hydrogen elimination providing an alkene function.

**Scheme 36:**
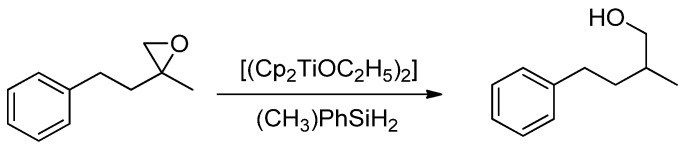
Reduction of epoxides mediated by [Cp_2_TiOC_2_H_5_)_2_]/(CH_3_)PhSiH_2_ system.

Very recently Gansäuer *et al.* have reported a new protocol to perform the radical reduction of epoxides by hydrogen atom transfer catalyzed by the active specie [Cp_2_TiH] [112]. The active catalyst is generated in situ from [Cp_2_TiOC_2_H_5_)_2_] in the presence of (CH_3_)PhSiH_2_ (Scheme 36).

#### 4.2.2. Intermolecular C-C Bond Formation

**Scheme 37:**
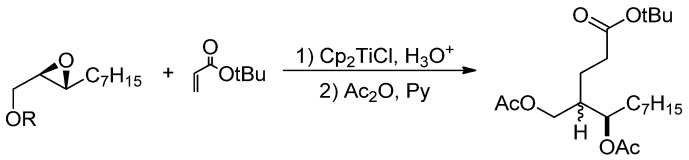
Intermolecular C-C addition reaction.

**Scheme 38:**
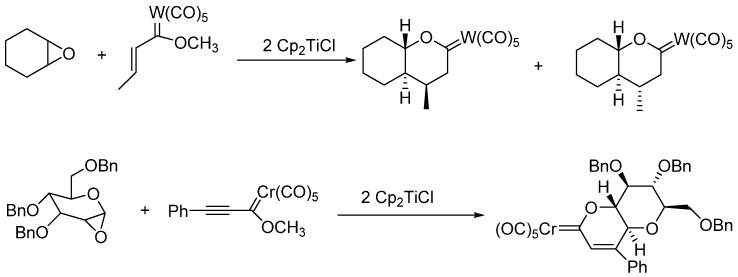
Addition reactions onto carbenes.

Epoxide-derived radicals generated in the presence of titanocenes are also intermediates for intermolecular C–C bond forming reactions [113]. The resultant products, like *δ*-hydroxyketones, esters, amides, nitriles and *δ*-lactones, constitute important classes of intermediates in organic synthesis (Scheme 37). It has been reported that the C–C bond formation employing *α*,*β*-unsaturated tungsten and chromium carbenes, as radical acceptors of epoxide-derived radicals, occurs with high chemoselectivity. This approach has been applied to both sugar-derived and *α*,*β*-unsaturated carbenes [114] (Scheme 38).

#### 4.2.3. Intramolecular C-C Bond Formation

**Scheme 39:**
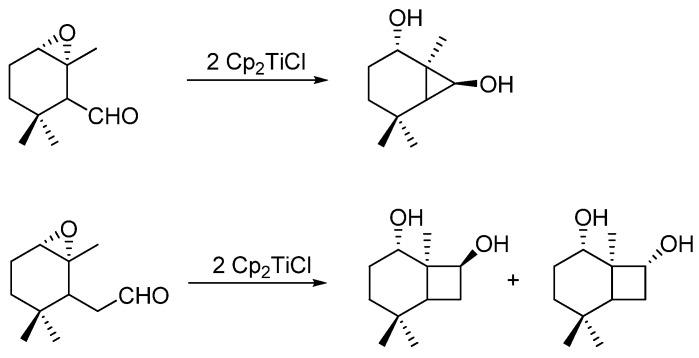
3-*exo* and 4-*exo* cyclization with aldehydes as radical traps.

The use of Cp_2_TiCl (in stoichiometric or catalytic amounts) allowed to promote the synthesis of natural products by the construction of three- to six- and eight-membered carbocycles in good to excellent yields [115,116,117,118,119,120]. Small ring 3-*exo* and 4-*exo* cyclizations were achieved by nucleophilic intramolecular radical addition to aldehydes and ketones [117] and natural products, like (E)-*endo*-bergamoten-12-oic acids [115] or carbacephams, are significant examples of synthetic applications (Scheme 39) [121,122,123,124,125].

**Scheme 40:**
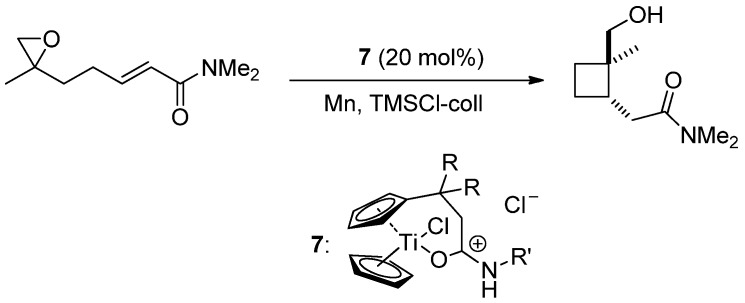
4-*exo*-cyclization of unsaturated epoxides.

**Figure 3:**
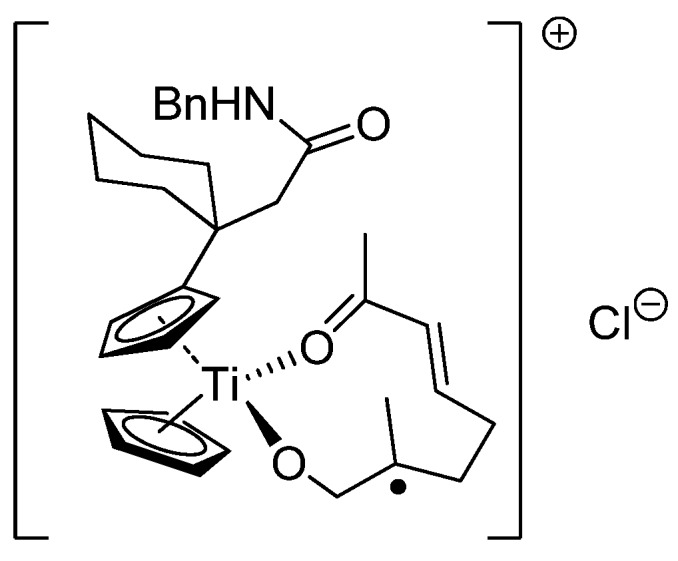
Possible radical intermediate in 4-*exo*-cyclization of unsaturated epoxides.

While *gem*-dialkyl or gem-dialkoxyl substitution is usually required to maintain an efficient propagation of the radical chain, very recently Gansäuer *et al.* have reported the first example of 4-*exo* cyclization on unsaturated epoxides without *gem* disubstitution [124]. This result was achieved by using cationic functionalized titanocenes as template catalysts (Scheme 40). According to the proposed mechanism [126], the two-point binding which occurs between the substituted radical and the template forces the radical center and the radical acceptor into close spatial proximity (Figure 3).

5-*exo* cyclizations are usually conducted by using alkenes and alkynes as radical traps and this approach has been widely applied for several synthetic routes [127,128,129,130,131,132,133,134,135,136,137]. Following an analogous procedure, a 6-*endo* cyclization employing Cp_2_TiCl was reported in 2001 by Takahaschi and co-workers for the synthesis of (±)-smenospondiol [138]. Titanocene(III) chloride mediated 5-*exo* and 6-*exo* cyclization has been recently applied for the regio- and diastereoselective synthesis of highly functionalized terpenic cyclopentanes [139], and for the synthesis of (−)-methylenolactocin and (−)-protolichesterinic acid [140]. Titanocene-promoted cyclization of unsaturated epoxylactones was also reported as a key step for diastereoselective synthesis of limonoid CDE fragments [141].

**Scheme 41:**
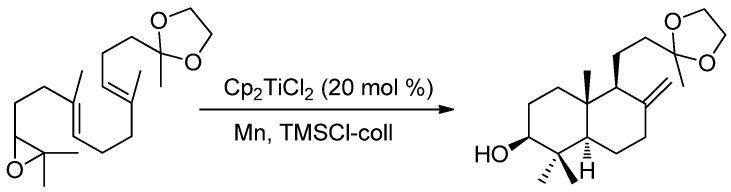
Catalytic epoxypolyene cyclization.

**Scheme 42:**
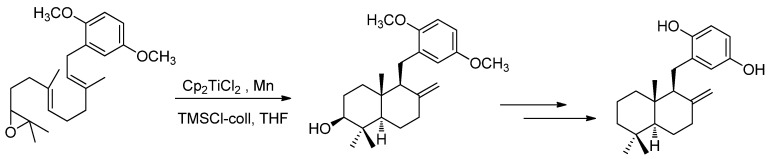
Tandem cyclization approach for meroterpenoid (+)-zonarol synthesis.

Epoxypolyene cyclizations were performed in the presence of Cp_2_TiCl_2_ using the collidine-chlorotrimethylsilane system, leading to exo-cyclic olefins by hydrogen atom abstraction (Scheme 41) [142,143,144]. These cyclization reactions proceed with high stereoselectivity giving only one of the many possible stereoisomers. Moreover, some of the cyclization products can be readily elaborated into natural products or key intermediates such as in the preparation of zonarol, a natural antifungal (Scheme 42).

**Scheme 43:**
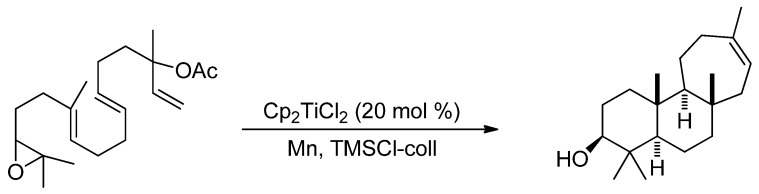
7-endo cyclization.

Following the same procedure, 7-*endo* cyclizations can be performed in surprisingly high yields (Scheme 43) [145]. Finally, 8-*endo* cyclizations have been reported by Roy and his group for the preparation of aromatic ethers [146].

## 5. Living Polymerizations

Several metal complexes were designed and tested to effectively control the radical polymerization of a number of polar olefins, leading to the synthesis of complex macromolecular architectures [147,148].

The use of titanium complexes for this purpose is relatively rare. Nevertheless, in recent years it has been emphasized the Ti(III)/Ti(IV) capacity of modulating the equilibrium in atom transfer reactions. As a consequence, this redox pair was reported as a very active catalytic system for atom transfer radical polymerization [149].

**Scheme 44:**
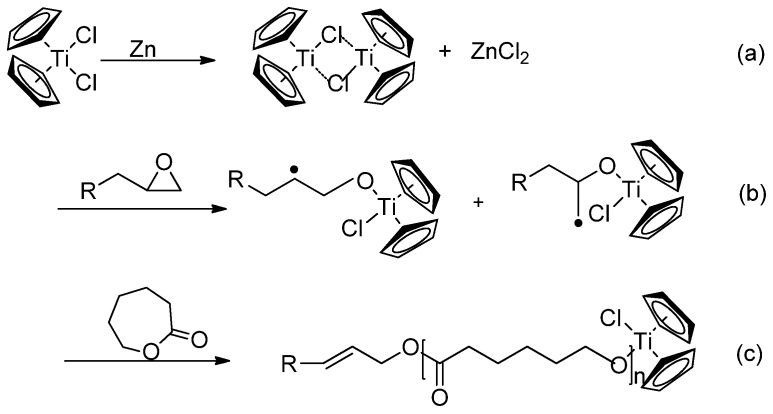
RRO of epoxides for the initiation of the living polymerization of e-caprolactone.

Radical controlled polymerization reactions by epoxide radical ring opening (RRO) have been performed in the presence of Cp_2_TiCl_2_ in combination with Zn powder [150,151,152]. According to this approach, methyl methacrylate (MMA) polymerization has been performed with high-molecular weight in aqueous conditions (Mw = 51,000–73,000). An analogous route has been recently reported for the radical polymerization of styrene [151]. The Ti(III)Cp_2_Cl-catalyzed RRO of epoxides produces Ti-alkoxides, which initiate the ring opening polymerization of cyclic esters like ε-caprolactone (Scheme 44) [153], the radical polymerization of isoprene [154] and styrene/isoprene copolymers [155].

The ligand effect in Ti-mediated living radical styrene polymerizations initiated by epoxide RRO has been widely investigated by Asandei and Moran, focusing in particular onto alkoxide and bisketonate titanium complexes [156], scorpionate and half-sandwich LTiCl_3_ derivatives [157] and substituted sandwich metallocenes [158]. Moreover, the same research group disclosed in detail the role of solvents and additives for this polymerization reactions [159], the effect of the reducing agent and temperature, as well as of the ratio titanium/epoxide and titanium/zinc [160].

Excellent results in the living radical polymerization were also achieved by initiating the process via single electron transfer reduction of aldehydes promoted by Cp_2_TiCl [161]. This approach has been successfully applied also for the polymerization of ε-caprolactone [162].

**Scheme 45:**
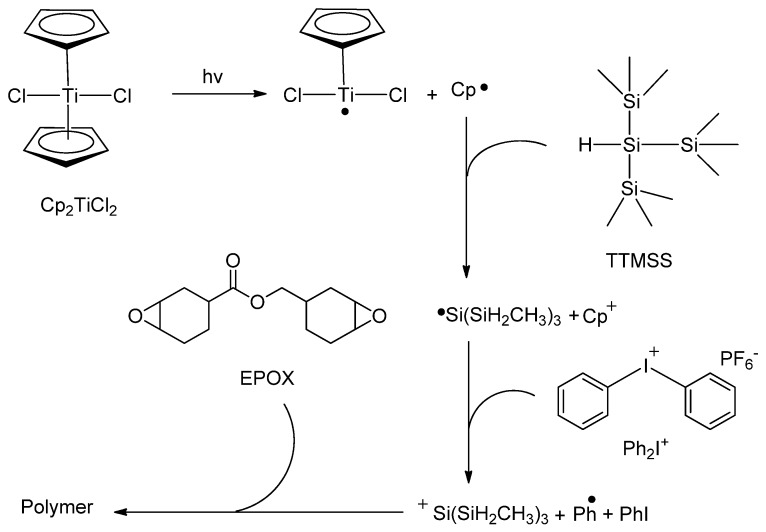
Polymerization of EPOX mediated by titanocene.

Very recently, Cp_2_TiCl_2_ has been applied in the field of photopolymerization for applications requiring safe conditions (Scheme 45) [163]. Cp_2_TiCl_2_/silane (tris(trimethylsilyl)silane TTMSS)/ iodonium salt (Ph_2_I^+^) resulted to be a good photoinitiating system in the free radical promoted cationic photopolymerization process of an epoxide monomer (EPOX) upon long wavelength excitation (λ > 400 nm). Under visible LED bulbs, xenon lamp and laser diodes, the reactions exhibit an excellent performance and a remarkable final polymerization close to 100%. The basic idea is to produce a radical (from a photoinitiating system) which, in turn, should be oxidized by the iodonium salt: Cp_2_TiCl_2_ generates under argon cyclopentadienyl radicals (Cp^•^) and/or metal-centered radicals CpTi^•^Cl_2_. In the presence of TTMSS, Cp^•^ can rapidly react by hydrogen abstraction with the silane Si-H generating a silyl radical. The latter can be easily oxidized by Ph_2_I^+^ with a rate constant of 2.6 × 10^6^ M^−1^ s^−1^, leading to the formation of a silylium cation, which, in turn, can initiate the cationic polymerization by ring-opening of EPOX.

## 6. Conclusions

This study covers the literature over the past twelve years emphasizing the key role that titanium salts and complexes play in promoting synthetic free-radical processes. While several excellent reviews, focusing on specific Ti-mediated reactions and defined product targets or on the general role of transition metals in promoting radical chains, have been recently published, to the best of our knowledge this work represents the first comprehensive contribution which outlines the potential of titanium catalysis in radical reactions, analytically overviewing the recent progresses in selective free-radical organic synthesis.

Though multicomponent procedures are of great interest for the development of new synthetic routes, examples in the field of radical reactions are in general scant. Among the few protocols recently reported, the nucleophilic radical addition to imines mediated by Ti(III)/Ti(IV) systems represents an innovative one-pot approach leading to a wide range of intriguing functionalized derivatives under very mild experimental conditions.

Pinacolization of aldehydes is particularly attractive, not only for the importance of the final diols, but also because it represents a significant model for investigating enantioselectivity in radical chemistry. Other radical coupling reactions, when mediated by titanium complexes, lead to high added value products with chemo- and stereo-selectivity.

Finally, deoxygenation or reductive ring opening of epoxides by means of titanocenes find ample use in the synthesis of natural and biologically active molecules. Ti-mediated radical ring opening of epoxides is also successfully applied to promote living radical polymerization of olefins (such as styrene and isoprene) and lactones (for example ε-caprolactone).

The development and optimization of titanium ligands, including the introduction of chiral complexes, allows in many cases to perform stero- and/or enantio-selective syntheses of the desired products. The ongoing research for the design, synthesis, and application of these new ligands guarantees the development of new Ti-based catalytic systems for selective transformations under very mild conditions.
